# *Artemisia vestita*: A Folk Medicine with Hidden Herbal Fortune

**DOI:** 10.3390/molecules28062788

**Published:** 2023-03-20

**Authors:** Shivani Dogra, Joginder Singh, Bhupendra Koul, Dhananjay Yadav

**Affiliations:** 1 Department of Microbiology, Lovely Professional University, Phagwara 144411, Punjab, India; 2 Department of Biotechnology, Lovely Professional University, Phagwara 144411, Punjab, India; 3 Department of Life Sciences, Yeungnam University, Gyeongsan 38541, Republic of Korea

**Keywords:** *Artemisia vestita*, traditional medicine, ethnopharmacology, phytoconstituents, cytotoxicity

## Abstract

Traditional medicines are nature’s gift and our native heritage, which play a vital role in maintaining a disease-free life. *Artemisia vestita Wall*. *ex Besser* (family: Asteraceae), popularly known as “Kubsha” or “Russian wormwood”, is a highly enriched folklore medicine with wound- healing, antiphlogistic, antifebrile, antifeedant, anti-helminthic, antimicrobial, antiviral, antitumor, and antiproliferative potential attributed to the presence of various volatile and non-volatile secondary metabolites. A systematic and extensive review of the literature on *A. vestita* was carried out via the Web of Science, PubMed, INMEDPLAN, EMBASE, Google Scholar, and NCBI, as well as from several websites. The highly relevant literature contained in 109 references was selected for further inclusion in this review. A total of 202 bioactive compounds belonging to different chemical classes such as terpenoids, coumarins, flavonoids, alkaloids, acetylenes, tannins, carotenoids, and sterols have been reported in *A. vestita*, which are responsible for different pharmacological activities. The chemical structures obtained from the PubChem and Chem Spider databases were redrawn using the software Chem Draw^®^ version 8.0. This review paper summarizes the distribution, botanical description, phytochemistry, pharmacological activities, and conservation of *A. vestita,* which will assist scientists for further investigation. Extensive studies on the active constituents, pharmaceutical standardization, mode of action, and sustainable conservation of *A. vestita* are needed to further explore its wound-healing and allied medicinal properties.

## 1. Introduction

The world is enriched with a treasure trove of traditional medicinal herbs that are of global importance for health security. India harbours four mega-biodiversity hotspots and is highly enriched with 17,500 species of medicinal plants used effectively against multiple disorders [1,2,3]. These medicinal herbs have been time-tested and recommended by saints, maharishis, vaidyas, and ayurvedic acharyas and have strong credence in different traditional medicinal systems such as ayurveda, unani, siddha, homeopathy, naturopathy, allopathy, and traditional Chinese medicine for treating ailments related to both humans and animals. Moreover, these medicines are safe, heal the cause of the ailment, and have less or no side effects compared to allopathic drugs [4]. For centuries, plant essential oils have played a provocative role for mankind. The people of Egypt were known to be skilled perfumers and taught the art of perfumery to Hebrews around 5000 years ago [5].

Earlier naturalized or wild plants provided social security to people in the form of supplements, fuel, fodder, raw material for companies, and an additional good income source. According to the WHO, approximately 80% of people are reliant on herbal remedies in developing nations. A total of 90% of herbal species used in India are brought from the western Himalayas, which is well known for its rich plant diversity, with 1748 medicinal species used in various fields such as pharmacological research, chemistry, clinical therapeutic studies, and pharmacognosy. Unfortunately, the traditional knowledge of herbal medicine is declining due to dependence on allopathy, which is associated with side-effects or ill effects on non-target organs. The synergistic effect of phytochemicals has multiple modes of actions that heal the disease and have immunomodulatory effects. In this post-COVID-19 era, people have become more conscious about their health and peace of mind. Thus, there is a drastic increase in interest and demand towards herbal medicines for improved quality of life [6]. The volatile aromatic oils find applications and are used commercially in cosmetics, soap, perfumery, the spice industry, flavoured tea, drinks, traditional foods, pesticides, and pharmaceutical industries [7]. The use of herbal-origin essential oils has increased greatly over the last few years, which has consequently increased the international market demand and decisively switched the trade. Sharma et al. [8] reported that India produces 15 essential oils on a pilot/experimental scale and approx. 20 herbal essential oils at a commercial level that have a unique capability, and India holds an economic advantage due to its rich diversity in soil and favourable climate. The oil content and quality depend on the type of soil and the climatic conditions.

*Artemisia* is a medicinally important genus belonging to the Asteraceae family which is also known as the Compositae family, thistle family, daisy family, and sunflower family [9]. This genus is gaining much attention due to its remarkable medicinal properties, phytochemical diversity, and scientifically proven health benefits [10,11,12,13,14].

The Chinese knew the therapeutic value of *Artemisia* 2000 years ago. In 1596, for the treatment of malaria symptoms, Li Shizhen suggested tea made from *Artemisia* (qinghao). The name ‘Artemisia’ was eventually derived from the great Greek goddess (Roman Diana) Artemis, the name of the Greek queens *Artemisia* I and II [15], and specifically named after the Caria Queen *Artemisia* II, who was a medical researcher and botanist by profession in the 4th century BC. The genus *Artemisia*, commonly known as wormwood, mugwort, or sagebrush, is distributed in the temperate zones of Europe, North America, and India [16]. Species of this genus are used as folklore medicines and in pharmaceutical sectors their demand has increased because of their medicinal value and high commercial importance [17]. The genus *Artemisia* comprises 500 species [18,19]. The greatest number of species seems to occur in Asia, with 174 spp. in the ex-USSR, 150 spp. in China, 50 spp. in Japan, 35 spp. in Iran, and 35 spp. in India, mostly from the northwestern Himalayas [20,21]. In India, the eminently recognized species that have been used traditionally are *Artemisia vestita*, *Artemisia dracunculus*, *Artemisia brevifolia*, *Artemisia roxburghiana*, *Artemisia dubia*, *Artemisia herba-alba*, *Artemisia japonica*, *Artemisia santolinifolia*, *Artemisia maritima*, *Artemisia scoparia*, *Artemisia absinthium*, *Artemisia verlotiorum*, *Artemisia maritima*, *Artemisia annua,* and *Artemisia vulgaris.* Mucciarelli and Maffei [22] reported that Artemisia possess antioxidant, antimicrobial, anticoagulant, antispasmodic, antidiabetic, anti-helminthic, anticancer, anti-ulcer, anticonvulsant, stomachic, cardiac stimulant, insecticidal, febrifuge, and cytotoxic properties and are also used for the treatment of coughs, colds, dyspepsia, headaches, malaria, and inflammation [23]. It is quite difficult to differentiate among the *Artemisia* species due to their morphological similarity [24], which leads to misinterpretation and misidentification of the products that are of economic and commercial medicinal value [25]. Several Artemisia-based health care products (tablets, syrups, oils, creams) have been commercialized.

The World Flora Online [26] reported *A. vestita* Wall. ex Besser as an accepted botanical name, whereas *A. vestita* Wall. ex DC. and *A. vestita* var. *vestita* are synonyms of *A. vestita.* In India, one of the major aromatic plants, i.e., *A. vestita,* is under commerce, which is obtained by distillation, hydro diffusion, expression, solvent extraction, and the natural carriers–host organisms [7]. Traditionally, local farmers and tribal communities refer to *A. vestita* as an anti-helminthic agent because of the unavailability and high cost of synthetic drugs [27,28]. It is widely used by tribal people in the Kashmir Valley (‘Tethe-Ven’) for treating parasitic infections [29]. In Tibet, it is commonly known by the name ‘Maolianhao’ (alias: Wannianpeng), a folk medicine [30], whereas in Kashmir it is known as ‘Roosi Tyethven’ [31]. It is also commonly known as ‘Russian Wormwood’, ‘Ganga Tulsi’, ‘Buer’, ‘Drubsha’, ‘Seski, Kubsha’, ‘Chamariya’, ‘Kundja’, and ‘Kundiyaa’.

To date, there have been fragmentary reports on *A. vestita* extracts, their mode of action, standardization, dose optimization, and toxicity. Through this article, we have tried to bridge the gap and provide explicit information on the distribution, botanical description, phytochemistry, and pharmacological activities of Russian wormwood.

## 2. Distribution

**Figure 1:**
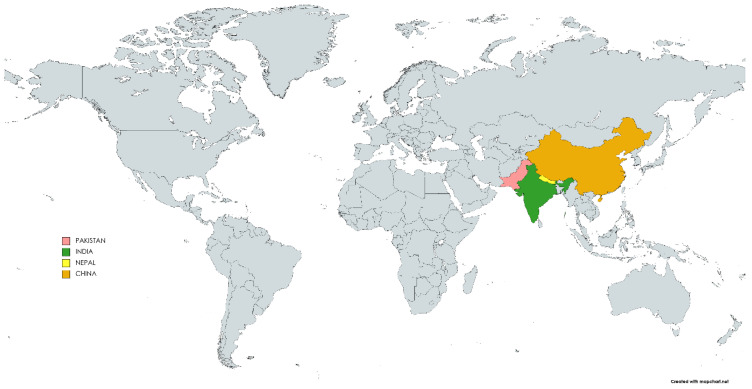
World map showing the distribution of *A. vestita* (coloured part showing regions).

*Artemisia vestita* is widely distributed in East Asia including the Himalayas of Nepal, Pakistan, India, and Tibet to southern and central China (Figure 1), including hills, rocky slopes, grasslands, shrublands, and outer forest margins of various regions of Gansu, northwest Guangxi, north Hubei, Guizhou, Liaoning, west Sichuan, Qinghai, Xizang, Xinjiang, Yunnan, north India, Nepal, and north Pakistan. Due to geographical variation and seasonal factors, drastic variations have been reported in the chemical composition of the essential oil. In India, Drubsha is found in Himachal Pradesh, Kashmir, and Uttarakhand at an altitude of 2100–3000 m. The distribution of *A. vestita* in the Pooh region of Kinnaur district at different elevations noted a density of 1.27/ha, frequency of 10%, abundance of 12.67, ratio of abundance to frequency (A/F) of 1.27, and importance value index of 5.12 at an elevation of 2700–3200 m; a density of 2.25/ha, frequency of 15%, abundance of 15, A/F of 1, and IVI of 7.28 at an elevation of 3200–3700 m; and a density of 1.17/ha, frequency of 11.67%, abundance of 10, A/F of 0.86, and IVI of 53.53 at an elevation of 3700–4200 m [32]. Important value indexes are the sum of the frequency, density, and dominance of the individual species.

## 3. Botanical Description

### 3.1. Morphology

**Figure 2:**
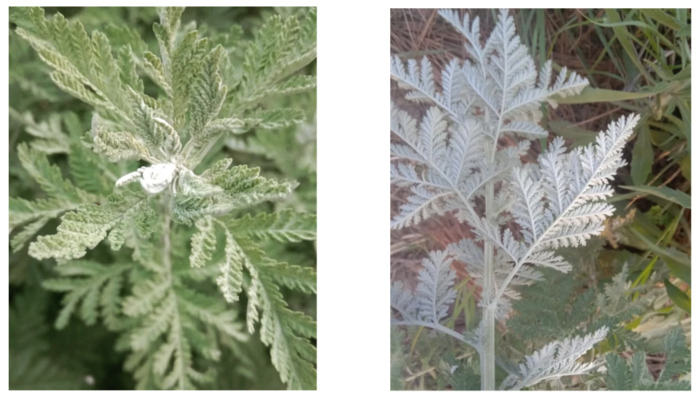
Leaves of *A. vestita* (adaxial (**Left**) and abaxial surface (**Right**)).

*A. vestita Wall*. *ex Besser* is an aromatic, erect, perennial shrub that may attain a height of 2 m (5–120 cm) [33]. The leaves are fern-like, soft, and hairy on the upper surface white and hairy towards the lower side, and pinnately cut. The flowers are small and appear creamy yellow, arranged in racemes (6–10); the heads of flowers are long, hairy, and compound, hanging gracefully on their slender nodding stalks. The fruits are shiny and smooth and the bracts are oblong and membranous (Figure 2). The leaves have clear abaxial and adaxial surfaces. Adaxial surface-elongated epidermal cells are partitioned and surface grooved with prominently ridged margins; the tertiary sculpture is aggregated; stomata are depressed, surrounded by thick, flat peristomal rims, transverse striata, thick inner ledges, and a gradually concave surface of the guard cells and transverse polar folds. The lobule tips are cap-like and swollen; stomata are also present in this particular region, with cells greatly undulated (V-shaped undulations) and elongated. The abaxial surface–cell outline is similar, somehow deeply undulate, with loose ‘V’-shaped undulations; it is tertiary sculptured and coarsely granular; the guard cells have oblique folds [34,35,36].

### 3.2. Vegetation Details

*A. vestita* can dominate the grassland ecosystem [37]. The *A. vestita* plants are hermaphrodite and pollinated by insects, and the seeds ripen in the months of August–October.

*Habitat*: Woodland edge garden, sunny position, cultivated beds, hills, rocky slopes, grasslands, shrublands, and exterior forest margins at an altitude of 2000–4300 m above sea level [38]. *Cytology*: Gupta et al. [39] reported 2*n* = 2x = 36 meiotic chromosome count, ploidy level (4×), pollen fertility 78–82%, and pollen grain size 22–24 μm in *A. vestita* collected from Haripurdhar and Churdhar (HP) at an altitude of 2400 and 3650 m, respectively, above sea level. Two other cytotypes, i.e., hexaploid (2*n* = 54) [40] and diploid (2*n* = 18) [41], have also been reported. *Chlorophyll content*: Variations in the chlorophyll and anthocyanin content and the chlorophyll/carotenoid ratio have been reported in the temperate species of *A. vestita* found at an altitude of 550 and 3600 m above sea level in the Garhwal Himalayas. The total chlorophyll content observed in the lower leaves of *A. vestita* was 1.716 and 1.470, middle leaves 0.902 and 1.650, and top leaves 0.863 and 1.205 at two different altitudes (550 and 3600 m, respectively) [42]. The molar chlorophyll/carotenoid ratio was observed to be lower (1) in the temperate species as compared to the tropical (1.7) and subtropical species (1.3) [43]. At higher altitudes, *A. vestita* plants have relatively broader adaptability potential compared to lowland species, which is due to a higher osmotic concentration, greater lignification, and the tendency of osmoregulation in tissues, due to the conversion of starch into sugar content [44]. A palynological study of *A. vestita* stated the quantitative characteristics of the plant, such as a polar axis of 19.38 ± 1.52µm, equatorial axis of 18.09 ± 1.51 µm, P/E (sphericity) of 1.07, thickness of exine of 2.13 ± 0.67 µm, and colpus length of 11.81 ± 1.69 µm, and spinules are prominent in the plant [45].

## 4. Medicinal Uses

*A. vestita* has been used as a folkoric medicine and was harvested from wild forests for use in anti-inflammatory and antifebrile medicines. Both aqueous and alcoholic solvents give a maximum amount of medicinal extract from the plant compared to other solvents [46]. It is widely used for treating numerous inflammatory diseases in Tibet and China, such as contact dermatitis, rheumatoid arthritis, and sepsis [47,48]. The leaves are crushed and applied externally on the skin as hemostatic [49,50]. The plant is also used in treating stomach-aches [51].

## 5. Phytochemistry

The essential oils of Kubsha are volatile and complex mixtures of sesquiterpenes providing a strong odour to the herbal plant. Extraction is performed using steam distillation or hydro-distillation methods. Leaves, stems, barks, aerial parts, inflorescences, whole plants, fruits, seeds, flowers, and roots are used for the extraction of essential oil and further used to combat human ailments, but the composition varies due to altitudinal variation. The isolated compounds have been identified using various techniques such as GC-MS, GC-FID, HRMS, UV, IR FTIR, HPLC-MS, GLC-MS, HPLC, UPLC-ESI-QqQLIT-MS/MS, 1D and 2D NMR, X-ray crystallography, and silica gel and polyacrylamide chromatography. On the basis of a literature survey, it has been reported and evidently showed that Russian wormwood essential oil composition is greatly influenced by the climate or geographical region and exhibits remarkable chemodiversity. Phytochemical studies revealed that *A. vestita* contains several monoterpenes, flavones, and sesquiterpenoids, among which the camphor/eucalyptol chemotype appears predominantly in most of the Artemisia species [52,53,54]. A Chinese research group reported the isolation of 15 chemical compounds from *A. vestita* and identified them as taurin, isoferulic acid, 8-dimethoxy flavone, yomogin, friedelin, beta-sitosterol, α and β-amyrin, daucosterol, 7-hydroxy-6,8-dimethoxy coumarin, scoplatin, caffeic acid [55]. Camphene, 1,8-Cineole, thujone, camphor, artemisia ketone, caryophyllene, and germacrene D were reported as major components of Artemisia species essential oil [56]. Zhengming et al. [57] conducted preliminary research on the chemical compounds, which were identified as saponins, organic acids, tannins, phenols, anthraquinones, flavonoids, lactones, coumarin, alkaloids, volatile oil, triterpenes, or steroids. Another study found 12 chemical constituents from essential oil including six monoterpenic derivatives, three monoterpenes, and three sesquiterpenes, among them 1, 8-cineol, camphor, and borneol, which were 39.01%, 26.92%, and 19.23%, respectively [58]. In addition, daucosterol, stigmasterol, scopolin, scoparone, umbelliferone, and isoscopoletin-O-glucoside were yielded from the plant [59]. A sesquiterpenoid Allohimachalol was identified in ref. [60]; α-, β- and γ-himachalene, germacrene D, caryophyllene, himachalol, α- and γ-atlantone, *allo*-himachalol, 1,8-cineole, santolina alcohols and their acetates, yomogi alcohol, thujanols and thujones were reported in ref. [61]; yomogi alcohol, (E)-2,5,5-Trimethylhepta-3,6-dien-2-ol, alpha-Atlantone, Himachalol, and gamma-Himachalene were found in ref. [50]; alpha-terpinene, terpenyl acetate, thujyl alcohol, α- and β-phellandrene, nerol, cineol, thujyl acetate, neral, artemisol, and beta-thujone were reported in ref. [62]; and arvestonol, arvestolides D-J, and arvestonates A-C occurred in ref. [63]. Numerous sesquiterpenoids exhibiting biological activities were isolated from *A. vestita* [30,64] and had a great influence on the plant’s defense against phytopathogenic fungi and pests, so they can be further utilized as antifungal agents and natural insecticides (scopoletin, ruin, luteolin, salicylic acid, naringenin, eugenol, kaempferol, dihydroartemisinin, isoeugenol, artemether, chrysin, and artemisinin) [65,66,67]. Rutin compound was highest in the ethanolic extract of *A. vestita* collected from Jammu and Kashmirregion. In total, 27 compounds were identified, out of which the main components were eucalyptol, 1,8-cineol, grandisol, camphor, and germacrene D [68]; allohimachalol, himachalene, germacrene D, caryophyllene, and himachalol [69]; eucalyptol camphor and borneol [70]; 1,8-cineole, (E)-citral (13.7%), limonene, α-phellandrene, camphor, and (Z) and (E)-thujones [71]; β-caryophyllene, artemisia alcohol, artemisia ketone, 1,8-cineol, and α-phellandrene [70]; and pectolinarigenin, apigenin, cirsilineol, 5,7,3′,4′-tetrahydroxy-6,5′-Dimethoxyflavone, 7-methoxycoumarin, patuletin, annphenone, umbelliferone, scopoletin, 2,4-dihydroxy-6-methoxyacetophenone, and quercetin [72]. Flavones such as pectolinarigenin, cirsilineol, jaceosidin, cirsimaritin, quercetin, hispidulin, 6-methoxytricin, apigenin, and acacetin have reported from Lhasa, Tibet [14]. n-carpryaldehyde, a-phellandrene, 1,8-cineol, a-terpinene, thujone, thujyl alcohol, citronellol, citral, geraniol, aromadendrene, cadinene, and chamazulene have also been reported [73]. A total of 18 components was found in Daksum, Kokerrnag, Kashmir, out of which the principal components were (E)-citral, 1,8-cineole, limonene, camphor, α-phellandrene, and (Z) and (E)-thujones. The dominant group were oxygenated monoterpenes, comprising 73.1% of terpenes in the plant essential oil composition, followed by monoterpene hydrocarbons (17.3%) [71]. A higher content of oxygenated monoterpenes in plants imparts a strong characteristic aroma to the *Artemisia* species. α-amyrin, daucosterol, stigmasterol, β-sitosterol, scoparone, scopolin, umbelliferone, and isoscopoletin-o-glycoside have been extracted from the aerial parts of *A. vestita* [74]. Sesqueterpenoids found in the plant are known for cytotoxic, antiviral, and antiphlogistic activities [75,76,77,78].

β-caryophyllene, artemisia alcohol, artemisia ketone, 1, 8-cineol and α-phellandrene were found to be major active compounds in accessions from Nainital, Uttarakhand, whereas α-Pinene, Camphene, Artemiseole, α-Pinene, α-Myrcene, 1,8-Cineol (Eucalyptol), δ-Terpinene, Camphor, trans-Pinocamphone, a-Pinocarvone, Terpinen-4-ol, Grandisol, γ-Pyronene, Copaene, β-Cubebene, α-Caryophyllene, Caryophyllene, α-Amorphene, γ-Himachalene, Germacrene D, Aromadendrene, α-Zingiberene, γ-Elemene, δ-Cadinene, Caryophyllene oxide, α-Bisabolol oxide B, and (Z)-α–Santalolare active compounds were found in accessions from Mentougou District, Beijing. Pectolinarigenin, apigenin, cirsiliol, 7-methoxycoumarin, patuletin, annphenone, umbelliferone, scopoletin, 2,4-dihydroxy-6-methoxyacetophenone, quercetin, cirsilineol, jaceosidin, cirsimaritin, hispidulin, 6-methoxytricin, and acacetin were found in accessions from Lhasa, Tibet. Scopoletin, rutin, luteolin, salicylic acid, naringenin, eugenol, kaempferol, dihydroartemisinin, isoeugenol, artemether, chrysin, artemisinin, Himachalene, germacrene-D, caryophyllene, allohimachalol, himachalol, atlantone, yomogi alcohol, 1, 8-cineole, santolina alcohol, thujones, and thujanols were found in accessions from Srinagar, Kashmir. α-amyrin, daucosterol, stigmasterol, β-sitosterol, scoparone, scopolin, umbelliferone, isoscopoletin-o-glycoside, arvestonol, arvestolides D–J, and arvestonates A–C were found in accessions from China.

### Essential Oil

**Figure 3:**
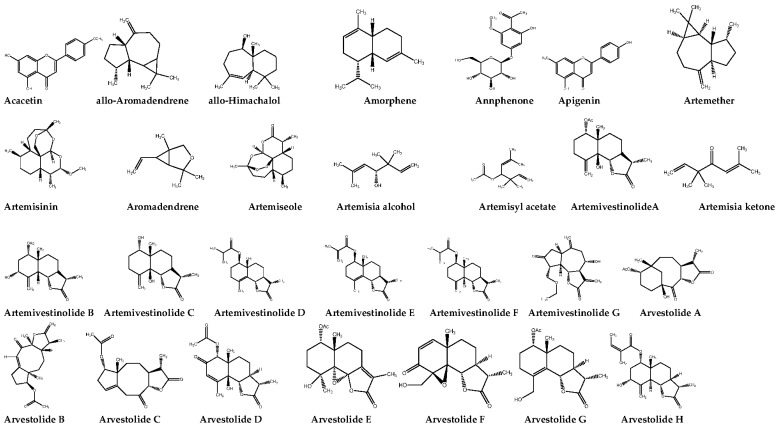

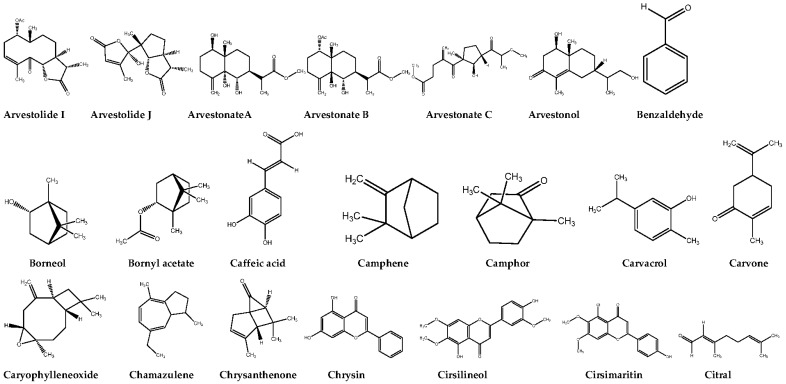

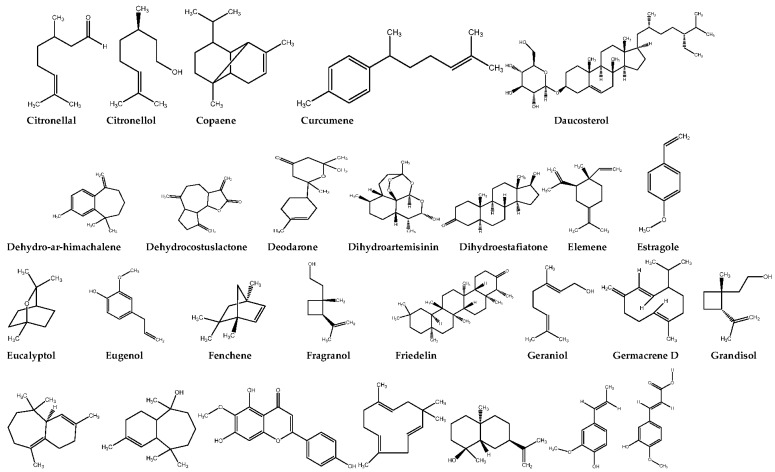

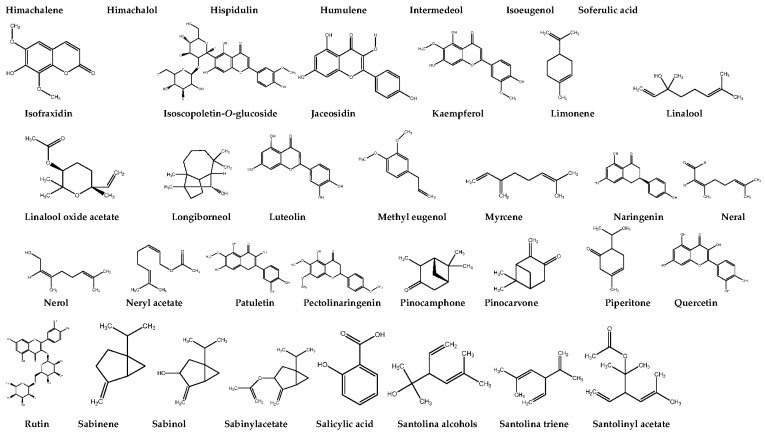

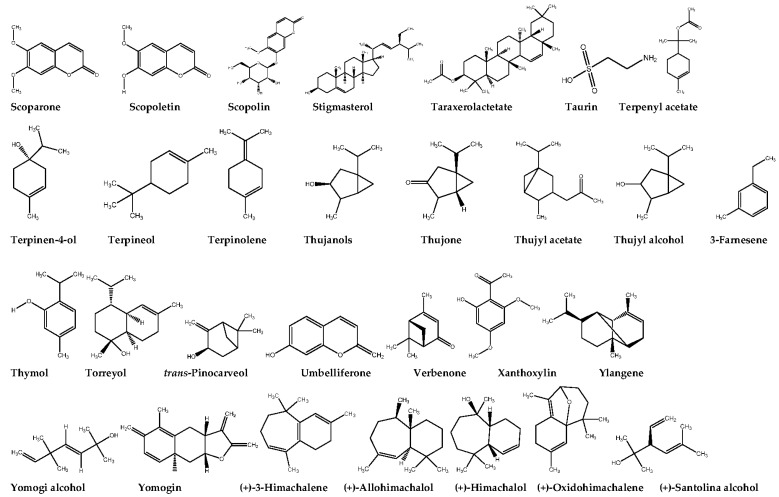

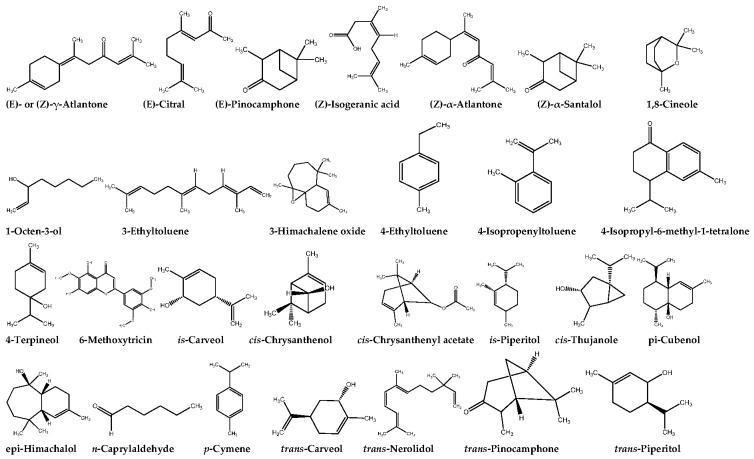

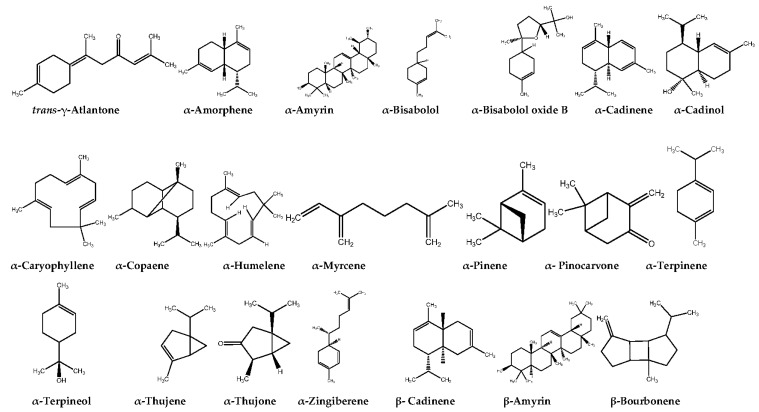

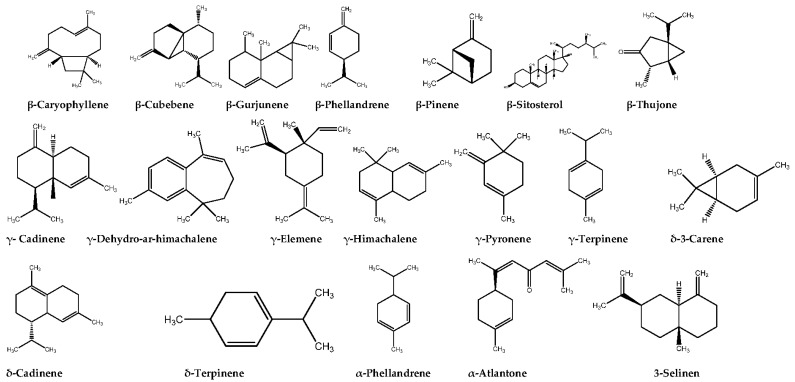
The chemical structures of bioactive compounds present in *Artemisia vestita*.

A total of 202 biochemical compounds have been reported from different parts (stem, leaves, roots) of *A. vestita*. There are mainly flavonoids, terpenoids, oxygenated monoterpenes, sesquiterpene lactones, oxygenated sesquiterpenes, sesquiterpenoids, hydroxyl cinnamic acids, monoterpene hydrocarbons, azulenes, sesquiterpene hydrocarbons, sterols, phenylpropanoids, monoterpenoids, hydroxycoumarins, coumarins, flavonoid glycosides, organosulfonic acids, oxygenated triterpenes, and aromatic aldehydes. The chemical structures of the bioactive compounds present in Russian wormwood are shown in Figure 3. The characteristic oil odour is determined by the constituents of fresh-smelling thujone and eucalyptol (1,8-cineole) and by the woody bark and the sweet note of atlantone and himachalol compounds. The odour of the plant’s essential oil is mainly woody, herbaceous, fresh, slightly sweet, and reminiscent of the sage and balsamic odour and is reported to be effective against dermatophytes [79]. The oil can be used safely in perfumery, scented soaps, and cosmetics. Camphor, borneol, and eucalyptol are responsible for the pleasant aroma and are also known for their antifungal and antibacterial activities. The flowering tops of the plants when subjected to steam distillation yield yellow-, orange-, or brown-coloured oil with an aromatic, sweet-woody odour [70]. The essential oil was produced from *A. vestita* from the Nainital hills, Shimla hills, and Kashmir valley [80,81]. The physiochemical characteristics observed in the essential oil of *A. vestita* are a refractive index D of 1.4915, ester value of 55.45, acid value of 1.3, carbonyl percentage of 20.80, ester value of 124.4 after acetylation, 1:1 solubility (in 95% alcohol), and specific gravity of 0.910 [70].

## 6. Pharmacological Activity

Kubsha is highly enriched with various phytochemicals responsible for enormous pharmacological activities (Table 1) such as antiphlogistic, antifebrile, antifeedant, anti-helminthic, antibacterial, antifungal, antiviral, antitumor, antiproliferation, antidote, immunosuppressive activity, diuretic, hypoglycemic, antiepileptic, antioxidative, wound-healing, clearing away itching, ringworms, skin infections, and respiratory tract infections, ethnic therapy for colds, sinus drainage, maintaining ventilation, reducing inflammation and asthma, and many more. The anti-adipogenic activity of *A. vestita* requires in-depth studies [82].

### 6.1. Wound-Healing

Fresh leaf paste is applied on wounds or cuts to stop the bleeding and inflammation [89]. Chinese medicines include *A. vestita* as an alternative and complementary medicine for the treatment of skin diseases. *A. vestita* has a cold nature and is capable of treating skin eruptions, heat, and itching [90]. Pastes of the leaves are applied for the treatment of skin infections, inflammation, ringworm, wounds, respiratory tract infections, ethnic therapy for colds, sinus drainage, and asthma [14,91,92,93,94].

### 6.2. Antidote

*A. vestita* has been used for the treatment of snake bite due to its high content of monoterpenes, flavones, and sesquiterpenoids in the leaf extracts.

### 6.3. Antimicrobial

The *A. vestita* essential oils are mainly composed of an odoriferous mixture of sesquiterpenes, monoterpenes, and aromatic compounds, which are used in naturopathy are very well known for their antimicrobial properties. Two major compounds, namely, grandisol and 1,8-cineol, have shown in vivo and in vitro antibacterial activity against respiratory-infection-causing bacteria. The oil exhibited MIC (minimum inhibitory concentration) values between 20 and 80 µg/mL, whereas the constituents exhibited between 130 and 200 µg/mL. The in vivo studies showed significant results of the oil and its component grandisol, which did not produce any toxic effects in mice [83]. Eight components that have been reported to exhibit antibacterial activity are α- and β-thujone, terpinen-4-ol, linalool, nerol, geraniol, α-pinene, and 1,8-cineole; their percentages in the oil were determined in ref. [84]. The plant extract and formulated gel have also shown significant results, as the plant extract exhibited MIC values between 100 and 240 µg/mL while the formulated gel (extract + natural polymer) exhibited MIC values between 30 and 85 µg/mL (unpublished work) against skin-infection-causing bacterial and fungal species.

### 6.4. Immunosuppressive Activity

Plant constituents are useful in healing immunological disorders such as autoimmune disorders and also in organ transplantation, as they produce immune-suppressive agents [95]. It has been reported that the essential oil of plants with higher amounts of α- and β-thujone have lesser or trace amounts of eucalyptol and camphor [96]. Jaceosidin exerts immunosuppressive effects both in vitro and in vivo through the activation and inhibition of T-cell proliferation, which is associated with the down-regulation of interferon (IFN)- gamma signal transducers and activators of the (STAT1) transcription1 and transcription factor T-box TBX-21 signaling pathway [72]. In addition, flavones such as apigenin, cirsilineol, and 6-methoxytricin from *A. vestita* have shown immunosuppressive and anti-inflammatory effects [14]. These flavones specifically inhibit PCA (passive cutaneous anaphylaxis), which induces contact hypersensitivity, whereas lymphocyte proliferation is induced by Con-A and CD-25 expression in T-cells, which shows immunosuppressive effects.

### 6.5. Anti-Inflammatory Activity

*A. vestita* extracts exhibit anti-inflammatory activity such as degranulation inhibition in mast cells and inflammatory cytokine production [63]. The plant extracts inhibited the proliferation of mouse splenocytes and mixed lymphocytes while reducing the IL-2 interleukin level and the level of metallo-proteinase-9 in vivo and in vitro [14].

The LD_50_ of less than 1000 mg/Kg was devoid of antiprotozoal, antibacterial, antifungal, antiviral, anthelmintic, diuretic, hypoglycaemic and anticancer, which showed its effect on isolated guinea pig respiration, ileum, nictitating membrane, cardiovascular system, and central nervous system [50]. *A. vestita* extract cross-linked with tragacanth gum (crosslinked polyacrylic-acid-based hydrogel) showed anti-inflammatory activity. The inhibition exhibited by a plant extract and plant extract formulated gel with a natural polymer of COX-1 (Cyclooxygenase-1) was found to be 97.962 ± 0.892% and 69.812 ± 0.911%, respectively, at 500 µg/mL concentration. Similarly, our group also explored a significant inhibition of cyclooxygenase-2. The inhibition percentage by the plant extract and plant extract formulated gel were 89.47 ± 1.401% and 52.76 ± 1.110%, respectively (unpublished work).

### 6.6. Anti-Epileptic Activity

Hispidulin is a flavonoid naturally occurring in *A. vestita* with powerful anti-epileptic activity. Hispidulin showed a 21.1893 Kcal/mol in silico docking score while targeting the human enzyme glycogen phosphorylase-b/chrysin, which showed the greater potential of this inhibitor molecule to become an effective antidiabetic drug to control hyperglycemia in type-2 diabetes. However, the work demands thorough in vivo and in vitro studies for the molecules to be used as anti-hyperglycemic drugs [85,97].

### 6.7. Antifeedant Activity

The compound artemivestinolide showed antifeedant activities against the third-instar larvae of *Plutella xylostella* with EC50 values of 25.3–42 and against the phytopathogenic fungi *F. oxysporum* (MIC-256 mg/L), *P. oryzae* (MIC-128 mg/L), and *B. cinerea* (MIC-256 mg/L) [86]. *A. vestita* ethanolic extract exhibited anti-inflammatory, anti-helminthic, and insecticidal activity against *Haemonchus contortus* and *Sitophilus zeamais,* respectively [60,65,86]. The whole-plant extract containing vegetative shoot exhibited a 87.2% reduction in the faecal egg count at 100 mg/kg, which showed significant activity against adult worms and larvae after 28 days post-treatment [86]. In a study, essential oil of *A. vestita* showed potential fumigant activity with an LC_50_ value of 13.42 mg/L and LD_50_ value of 50.62 mg against adult *Sitophilus zeamais* in the fumigant bioassay and in the contact bioassay, respectively [65]. Several monoterpenes, sesquiterpenoids, and flavones have been isolated from *A. vestita* [20,72] and the essential oil chemical composition has been well-studied [69,70]. 1,8-cineol has a cold-relieving effect with mucolytic and expectorant properties [98]. 1,8-cineol and camphor present in plant essential oil act as fumigants with a broad insecticidal activity [99,100,101] and possess the potential to expand as a novel natural fumigant for insect control in stored products [102,103]. They are advantageous over conventional fumigants because they are non-persistent, biodegradable, and easily procurable and exhibit low toxicity to mammals [104].

## 7. Known Hazards

Although no specific reports on toxicity have been recorded for *A. vestita* extracts, the genus *Artemisia* contains allergenic sesquiterpenoid lactones that have the potential to cause skin reactions or dermatitis [105].

## 8. Cytotoxicity

The presence of volatile terpenoids and monoterpenes, i.e., pinene, eugenol, 1,8-cineole, limonene, citronellol, terpinolene, citronellal, thymol, and camphor, in *A. vestita* essential oil constituents provides repellent or toxic activity [106]. Compounds named Arvestolides H and I showed inhibitory effects in BV-2 cells on nitric oxide production, which was induced by lipopolysaccharide with an IC 50 value of 43.2 μM for Arvestolides H and 39.9 μM for Arvestolides I. [61], whereas cirsilineol, apigenin, and 6-methoxytricin are the active components that inhibited the proliferation of T-cells and the activation of in vitro bioassays. Immune-suppressive compounds extracted from *A. vestita* will be an effective remedy for T-cell-mediated inflammation [14]. Sesquiterpenes, coumarins, and flavones were reported in wormwood [107]. The aqueous leaf extract alleviates picryl chloride (PCl)-induced contact hypersensitivity by blocking the T lymphocyte activation [108]. In a study, flow cytometric and MTT assays were used for determining the CD 25 expression in T-cells and the proliferation of Con A induced lymphocytes [14]. For cytotoxic effects, both S2 (extract) and S4 (extract + polymer) showed higher cell viability. At a 1000 µg/mL concentration of S2, the cytotoxicity to HaCat cells was 18.2 ± 0.35% compared to that of S4, which was 19.7 ± 0.29%. The results showed promising anti-inflammatory effects along with significant anti-cancer effects on HaCat cell lines (unpublished work). The aforementioned results provide *A. vestita*-based folklore medicine a rationale to be used in wound-healing and anti-cancer therapy.

### 8.1. Biological Activity of Annphenone

Annphenone showed specific and potent antiproliferative activity against HepG2 cells and the IC50 value was 2.0 ± 0.4 μg/mL. During the cell cycle analysis in the G0/G1 phase, annphenone compound arrested the HepG2 cells when detecting the immunocytochemistry. It is suggested that the annphenone compound inhibits the catenin expression induced by the localization transfer, reducing the cyclin D1 protein expression. Furthermore, annphenone’s interaction as a possible ligand of the ASGP-R asialoglycoprotein receptor using a molecular docking simulation revealed its selectivity for hepatocellular carcinoma cells and potentially specific for antiproliferative activity [71]. Annphenone is a promising anti-tumour agent present in the aqueous extract that reduced the contact sensitivity via down-regulating the adhesion, activation, and production of metalloproteinase T-lymphocytes in mice [109], whereas the ethanolic extract exerted anti-sepsis activity through down-regulation of the NF-kB and MAPK pathways [63].

### 8.2. Biological Activity of Cirsilineol

Cirsilineol (4′,5-dihydroxy-3′,6,7-trimethoxyflavone) found in *A. vestita* extracts possesses potent anti-tumour and immune suppressive properties [14,87]. Cirsilineol significantly inhibited the proliferation of multiple types of cancer cells (Skov-3, PC3, Caov-3, and Hela cells) in a concentration-dependent manner. It induced apoptosis in Caov-3 cells in a dose-dependent manner, which was determined with annexin V/propidium iodide double staining. To promote apoptosis, cirsilineol activates caspase-9, caspase-3, and PARP (poly ADP-ribose polymerase). Cirsilineol-induced loss of the mitochondrial membrane potential (MMP) brings a remarkable change and releases cytochrome-c to the cytosol. The induction of apoptosis via the mitochondrial pathway is the mode of action for the anti-proliferative activity of cirsilineol against cancerous cells. Moreover, cirsilineol is effective in ameliorating TNBS (tri-nitrobenzene sulfonic acid) -induced experimental colitis in mice, possibly because of its novel immunoregulatory activities with selective inhibitor IFN-γ/STAT1/T-bet signaling in the colonic lamina propria CD4 + T-cells particular for Crohn’s disease [88].

### 8.3. Biological Activity of Jaceosidin

Regulation of the transcription activator and signal transducer (STAT 1) is being explored for the treatment of bowel diseases. However, few chemicals have been reported for the inhibition of STAT1/ IFN-g signaling for the treatment of Crohn’s disease. A natural compound, cirsilineol, isolated from the *A. vestita* plant significantly ameliorated TNBS (trinitro-benzene sulphonic acid)-induced T-cell-mediated mice colitis. It is closely associated with reduced auto-reactive T-cell activation and proliferation. Moreover, the action of anti-inflammatory and pro-inflammatory cytokines with cirsilineol therapy was found to increase the regulatory T-cell activity and decrease the effector Th-1 cell activity, as characterized by the up-regulation of TGF-b and IL-10 and down-regulation of IFN-g. Importantly, in the presence of a higher level of IFN-g the inhibition by compound cirsilineol of STAT1/IFN-g signalling seems reversible, suggesting that compound cirsilineol might be a potential candidate for the treatment of human inflammatory T-cell-mediated bowel diseases [88]. The flavone jaceosidin isolated from *A. vestita* showed an antiproliferation effect on several human cancer cell lines. It significantly reduced SKOV-3, PC3, HeLa, and CAOV-3 cell proliferation in a concentration-dependent manner, whereas CAOV-3 showed time-dependent inhibition, and apoptosis increased in CAOV-3 cells. Flavone induced the cleavage of PARP (poly ADP-ribose polymerase) and caspase-3 and increased the cleaved caspase-9 levels. It also elevated the cytochrome c level in the cytosol, which shows the antitumor property of jaceosidin [87].

## 9. Conclusions

Herbal flora is gaining much attention from scientists for the development of strategies and to know the therapeutic potential of novel herbal constituents to treat various health disorders. The information regarding the use of *A. vestita* as a folkoric medicine was mostly confined to the native inhabitants.

The phytochemicals reported in *A. vestita* belong to the chemical classes of flavonoids, terpenoids, oxygenated monoterpenes, triterpenes, sesquiterpenes, hydroxyl cinnamic acids, mono- and diterpene hydrocarbons, aromatic aldehydes, azulenes, sesquiterpene hydrocarbons, sterols, phenylpropanoids, monoterpenoids, coumarins, and organosulfonic acids.

The phenolic compounds present in the oil are responsible for the antioxidant activity, whereas flavones and sesquiterpenes exhibit anti-tumor and anti-inflammatory activity, respectively. The anti-inflammatory activity of *A. vestita* leaf extracts hhasave been time-tested.

*A. vestita* can also be a promising source of anti-COVID-19 remedies. The inverse correlation between the antiviral activity of artemisinin contents and total flavonoid contents is reported. Artemisinin singly or in combination with other components acts synergistically to block the post-entry viral infection. Moreover, essential oil and extract from the pre- and post-flowering stages of *A. vestita* have been reported to possess antifungal activity against phytopathogenic fungi and can be an effective drug against dermatophytes.

Although *A. vestita* has been used for the treatment of numerous ailments, standardization and extensive clinical studies shall facilitate optimizing the dose. Moreover, the plant is not yet extensively explored by the scientific community, as the successful reports related to the efficacy of its extracts are fragmentary and show variable results.

Research related to the development of *A. vestita*-based value-added products and the enhancement of the efficacy of the extracts by blending with other natural extracts is also required. Being a reservoir of phytochemicals, it is necessary to conserve this species for further research so as to gain the associated medicinal benefits for health security.

## Figures and Tables

**Table 1:** The pharmacological activity of phytochemicals reported in *Artemisia vestita*.

Isolated Compound/Extract/Methodology	Pharmacological Activity	Reference(s)
Eucalyptol and grandisol	Antimicrobial activity	[83]
Sesqueterpenoid Allohimachalol	Spasmolytic activity	[70]
Ethanolic extract	Antibacterial, antiprotozoal, antifungal, antiviral, anthelmintic, diuretic, hypoglycaemic, and anticancer	[48]
Essential oil	Antibacterial and antifungal in dilution	[62]
Essential oil	Immune-suppressive agents	[84]
Alcoholic extract	Antifeedant and antifungal activity	[85]
Alcoholic extract	Anti-helminthic activity	[86]
Essential oil	Insecticidal activity	[67]
Jaceosidin compound on human ovary cancer cells CAOV-3, SKOV-3, PC3, and HeLa cells	Antitumor	[87]
Annphenone compound on HepG2 cells (liver hepatocellular carcinoma)	Antiproliferative activity	[71]
Rutin on 3T3-L1 cells	Anti-adipogenic activity	[66]
Cirsilineol	Human inflammatory bowel diseases	[88]
Plant aerial part extract	Anti-inflammatory and immune suppressive effect	[14,63]

## Data Availability

Not applicable.

## References

[1] Ved D., Goraya G. (2008). Demand and supply of medicinal plants. Medplant ENVIS Newsl. Med. Plants.

[2] Kar A., Borthakur S. (2008). Medicinal Plants Used against Dysentery, Diarrhoea and Cholera by the Tribes of Erstwhile Kameng District of Arunachal Pradesh. Nat. Prod. Radiance.

[3] Suresh J., Rajkumar P., Archana T. (2011). Indian medicinal plants: A rich source of natural immuno-modulator. Int. J. Pharm..

[4] Chan K. (2003). Some aspects of toxic contaminants in herbal medicines. Chemosphere.

[5] Haq N., Chomchalow N., Henle H.V. (1995). Breeding and improvement of medicinal and aromatic plants. Medicinal and Aromatic Plants in Asia.

[6] Kotnis M.S., Patel P., Menon S.N., Sane R.T. (2004). Renoprotective effect of *Hemidesmus indicus*, a herbal drug used in gentamicin-induced renal toxicity. Nephrology.

[7] Lal R., Chanotiya C., Gupta P., Deepa B., Mishra A. (2020). Essential oils their quality assurance, management and stakes of CSIR-CIMAP (India): Past and future perspective. JMAPS.

[8] Sharma J.R. (1997). Medicinal and Aromatic Plants in India: Current Status and Challenges Ahead. MAEER MIT Pune J. Spl..

[9] Koul B., Khatri T. (2020). The *Artemisia* genus: Panacea to several maladies. Bioactive Natural Products in Drug Discovery.

[10] Bora K.S., Sharma A. (2011). The genus *Artemisia*: A comprehensive review. Pharm. Biol..

[11] Tan R.X., Zheng W., Tang H. (1998). Biologically active substances from the genus Artemisia. Planta Med..

[12] Abad M.J., Bedoya L.M., Apaza L., Bermejo P. (2012). The *Artemisia* L. Genus: A review of bioactive essential oils. Molecules.

[13] Tian S.-H., Chai X.-Y., Zan K., Zeng K.-W., Tu P.-F. (2013). Three new eudesmane sesquiterpenes from *Artemisia vestita*. Chin. Chem. Lett..

[14] Yin Y., Gong F.-Y., Wu X.-X., Sun Y., Li Y.-H., Chen T., Xu Q. (2008). Anti-inflammatory and immunosuppressive effect of flavones isolated from *Artemisia vestita*. J. Ethnopharmacol..

[15] (2004). Encyclopædia Britannica Online. USA. https://www.britannica.com/.

[16] Wang W.-M. (2004). On the origin and development of *Artemisia* (Asteraceae) in the geological past. Bot. J. Linn. Soc..

[17] Kala C.P., Dhyani P.P., Sajwan B.S. (2006). Developing the medicinal plants sector in northern India: Challenges and opportunities. J. Ethnobiol. Ethnomed..

[18] Martín J., Torrell M., Korobkov A., Vallès J. (2003). Palynological features as a systematic marker in *Artemisia* L. and related genera (*Asteraceae*, *Anthemideae*)-II: Implications for Subtribe Artemisiinae delimitation. Plant Biol..

[19] Vallès J., Garnatje T. (2005). *Artemisia* and its allies: Genome organization and evolution and their biosystematic, taxonomic and phylogenetic implications in the *Artemisiinae* and related subtribes (*Asteraceae*, *Anthemideae*). Plant Genom. Biodivers. Evol..

[20] Greger H., Hofer O., Nikiforov A. (1982). New sesquiterpene-coumarin ethers from *Achillea* and *Artemisia* species. J. Nat. Prod..

[21] Rechinger K.H., Rechinger K., Hedge I.C. (1986). Artemisia in flora iranica. Compositae.

[22] Mucciarelli M., Maffei M. (2001). Introduction to the genus. Artemisia.

[23] da Silva J.A.T., Yonekura L., Kaganda J., Mookdasanit J., Nhut D.T., Afach G. (2005). Important secondary metabolites and essential oils of species within the Anthemideae (*Asteraceae*). J. Herbs Spices Med. Plants.

[24] Konowalik K., Kreitschitz A. (2012). Morphological and anatomical characteristics of *Artemisia absinthium* var. absinthium and its Polish endemic variety *A. absinthium* var. calcigena. Plant Syst. Evol..

[25] Muhammad A., Muhammad Q.H., Shazia J., Nighat S., Mir A.K., Ghazalah Y. (2010). *Artemisia* L. species recognized by the local community of the northern areas of Pakistan as folk therapeutic plants. J. Med. Plant Res..

[26] List The Plant. Version 1.1. http://www.theplantlist.org/.

[27] Iqbal Z., Lateef M., Ashraf M., Jabbar A. (2004). Anthelmintic activity of *Artemisia brevifolia* in sheep. J. Ethnopharmacol..

[28] Tariq K., Chishti M., Ahmad F., Shawl A. (2009). Anthelmintic activity of extracts of *Artemisia absinthium* against ovine nematodes. Vet. Parasitol..

[29] Ahad S., Tanveer S., Nawchoo I.A., Malik T.A. (2017). Anticoccidial activity of *Artemisia vestita* (*Anthemideae*, *Asteraceae*)—A traditional herb growing in the Western Himalayas, Kashmir, India. Microb. Pathog..

[30] Tian S., Chai X., Zan K., Zeng K., Guo X., Jiang Y., Tu P. (2013). Arvestolides A–C, new rare sesquiterpenes from the aerial parts of *Artemisia Vestita*. Tetrahedron Lett..

[31] Mochi S.A., Riyaz M. (2021). A preliminary survey of ethnomedicinal flora along pir panjal gradient (Kashmir-Himalayas), aharbalKulgam (J&K ut), India. J. Plant Dev. Sci..

[32] Verma R.K., Kapoor K.S. (2010). Assessment of Floristic Diversity in Pooh Valley of Cold Deserts of District Kinnaur, Himachal Pradesh. Biol. Forum Int. J..

[33] Putievsky E. (1991). Vegetative propagation of aromatic plants of the mediterranean region. Herbs Spices Med. Plants Recent Adv. Bot. Hortic. Pharmacol..

[34] Mehrotra S., Mehrotra B., Aswal B., Sharma H. (1990). Leaf surface studies of some medicinal artemisias. Int. J. Crude Drug Res..

[35] Jackson W.P.U. (1990). Origins and Meanings of Names of South African Plant Genera.

[36] WFO (2022). Artemisia vestita Wall. ex Besser. http://www.worldfloraonline.org/taxon/wfo-0000083111.

[37] Zeng Q., Liu Y., Xiao L., An S. (2020). Climate and soil properties regulate soil fungal communities on the Loess Plateau. Pedobiologia.

[38] Huxley A.J., Griffiths M. (1992). Dictionary of Gardening.

[39] Gupta R.C., Goyal H., Singh V. (2014). Cytology of the genus Artemisia (*Anthemidae*, *Asteraceae*) in the Western Himalayas. Biologia.

[40] Marhold K., Aguilera P.M., Daviña J.R., Honfi A.I., Bala S., Gupta R.C., Fader A.A.C., Cabral E.L., Gosavi K.V.C., Yadav S.R. (2011). IAPT/IOPB chromosome data 12. Taxon.

[41] Kaul M., Bakshi S. (1984). Studies on the genus *Artemisia* L. in North-West Himalaya with particular reference to Kashmir. Folia Geobot. Phytotaxon..

[42] Nautiyal S. (1986). High altitude acclimatization in Artemisia. Changes in chlorophyll contents. Ind. J. Pl. Physiol..

[43] Todaria N. (1986). Changes in pigments and total phenolics in Artemisia species grown at different altitudes in the Garhwal Himalaya. Biol. Plant..

[44] Nautiyal S. (1983). High altitude acclimatization in four Artemisia species: Changes in soluble sugars, starch and lignin contents in the leaves. Biol. Plant..

[45] Hayat M.Q., Ashraf M., Khan M.A., Yasmin G., Shaheen N., Jabeen S. (2010). Palynological study of the Genus Artemisia (*Asteraceae*) and its systematic implications. Pak. J. Bot..

[46] Dograa S., Singh J., Vashist H.R. (2021). Anthology of pharmacological activities from folklore medicine Artemisia. NVEO Nat. Volatiles Essent. Oils J..

[47] Nazemizadeh Ardakani P., Masoudi S. (2017). Comparison of the volatile oils of *Artemisia tournefortiana* Reichenb. obtained by two different methods of extraction. Trends Phytochem. Res..

[48] Qiangba C., Gama Q., Zhan D., Riren B. (2002). Zhong Hua Ben Cao, Volume of Tibetan Medicine.

[49] Dograa S., Singha J., Vashistb H. (2021). Extraction, isolation and pharmacognostical characterization of components from *Artemisia vestita* Wall ex Besser. NVEO Nat. Volatiles Essent. Oils J..

[50] Gupta A., Tandon N. (2004). Reviews on Indian Medicinal Plants.

[51] Pathak N., Karnick C. (1980). New folk-lore medicines from Sudh-Mahadeo region of Himalayas. Nagarjun.

[52] Lopes-Lutz D., Alviano D.S., Alviano C.S., Kolodziejczyk P.P. (2008). Screening of chemical composition, antimicrobial and antioxidant activities of *Artemisia* essential oils. Phytochemistry.

[53] Lemberg S. (1982). Armoise-Artemisia herba-alba. Perfum. Flavor.

[54] Perez-Alonso M., Velasco-Negueruela A., Palá-Paúl J., Sanz J. (2003). Variations in the essential oil composition of *Artemisia pedemontana* gathered in Spain: Chemotype camphor-1, 8-cineole and chemotype davanone. Biochem. Syst. Ecol..

[55] Yinjuan B., Yu L., Yanping S., Youhua H. (1997). Chemical constituents of *Artemisia vestita*. Zhongguoyaoxue Za Zhi Zhongguoyaoxue Hui 1989.

[56] Pandey A.K., Singh P. (2017). The genus *Artemisia*: A 2012–2017 literature review on chemical composition, antimicrobial, insecticidal and antioxidant activities of essential oils. Medicines.

[57] Zhengming Y., Bo L., Panpan L., Yijun C., Yuan L. (2014). Systematic preliminary research on the chemical components of *Artemisia vestita* Wall. Med. Plant.

[58] D Felicio J., B Soares L., C Felicio R., Gonçalez E. (2012). Artemisia species as potential weapon against agents and agricultural pests. Curr. Biotechnol..

[59] Agrawal P. (1990). Carbon-13 NMR spectroscopic studies of allohimachalol. Fitoterapia.

[60] Husain A. (1992). Dictionary of Indian Medicinal Plants.

[61] Tian S.-H., Zhang C., Zeng K.-W., Zhao M.-B., Jiang Y., Tu P.-F. (2018). Sesquiterpenoids from *Artemisia vestita*. Phytochemistry.

[62] Khare C.P. (2008). Indian Medicinal Plants: An Illustrated Dictionary.

[63] Sun Y., Li Y.-H., Wu X.-X., Zheng W., Guo Z.-H., Li Y., Chen T., Hua Z.-C., Xu Q. (2006). Ethanol extract from *Artemisia vestita*, a traditional Tibetan medicine, exerts anti-sepsis action through down-regulating the MAPK and NF-κB pathways. Int. J. Mol. Med..

[64] Kaul V.K., Nigam S.S., Dhar K.L. (1976). Antimicrobial activities of the essential oils of Artemisia absinthium Linn, *Artemisia vestita* Wall, and *Artemisia vulgaris* Linn. Indian J. Pharmacol..

[65] Wu H.-B., Wu H.-B., Wang W.-S., Liu T.-T., Qi M.-G., Feng J.-C., Li X.-Y., Liu Y. (2016). Insecticidal activity of sesquiterpene lactones and monoterpenoid from the fruits of *Carpesiumabrotanoides*. Ind. Crops Prod..

[66] Singh P., Bajpai V., Khandelwal N., Varshney S., Gaikwad A.N., Srivastava M., Singh B., Kumar B. (2021). Determination of bioactive compounds of *Artemisia* Spp. plant extracts by LC–MS/MS technique and their in-vitro anti-adipogenic activity screening. J. Pharm. Biomed. Anal..

[67] Chu S.S., Liu Q.R., Liu Z.L. (2010). Insecticidal activity and chemical composition of the essential oil of *Artemisia vestita* from China against *Sitophilus zeamais*. Biochem. Syst. Ecol..

[68] Weyerstahl P., Kaul V., Weirauch M., Marschall-Weyerstahl H. (1987). Volatile constituents of *Artemisia vestita* oil. Planta Med..

[69] Chowdhury A. (2003). GC/MS studies of volatiles from *Artemisia vestita* aerial parts. J. Essent. Oil Bear. Plants.

[70] Rather M.A., Dar B.A., Shah W.A., Prabhakar A., Bindu K., Banday J.A., Qurishi M.A. (2017). Comprehensive GC–FID, GC–MS and FT-IR spectroscopic analysis of the volatile aroma constituents of *Artemisia indica* and *Artemisia vestita* essential oils. Arab. J. Chem..

[71] Long A., Fu J., Hu Y., Luo Y. (2013). Annphenone from *Artemisia vestita* inhibits HepG2 Cell proliferation. Asian J. Chem..

[72] Yin Y., Sun Y., Gu L., Zheng W., Gong F., Wu X., Shen Y., Xu Q. (2011). Jaceosidin inhibits contact hypersensitivity in mice via down-regulating IFN-γ/STAT1/T-bet signaling in T cells. Eur. J. Pharmacol..

[73] Anibogwu R., Jesus K.D., Pradhan S., Pashikanti S., Mateen S., Sharma K. (2021). Extraction, isolation and characterization of bioactive compounds from *Artemisia* and their biological significance: A review. Molecules.

[74] Tan R., Lu H., Wolfender J.-L., Yu T., Zheng W., Yang L., Gafner S., Hostettmann K. (1999). Mono-and sesquiterpenes and antifungal constituents from *Artemisia* species. Planta Med..

[75] Kawazoe K., Tsubouchi Y., Abdullah N., Takaishi Y., Shibata H., Higuti T., Hori H., Ogawa M. (2003). Sesquiterpenoids from *Artemisia g Ilvescens* and an anti-MRSA compound. J. Nat. Prod..

[76] Wang B., Zhang G.-H., Yang Y.-F., Li P.-P., Liu J.-X. (2018). Response of soil detachment capacity to plant root and soil properties in typical grasslands on the Loess Plateau. Agric. Ecosyst. Environ..

[77] Ma C.-M., Nakamura N., Hattori M., Zhu S., Komatsu K. (2000). Guaianedimers and germacranolidefrom *Artemisia Caruifolia*. J. Nat. Prod..

[78] LaiBin Z., JiNao D., JieLi L. (2017). Phytochemistry and bioactivities of sesquiterpenoids from the *Artemisia* species. J. Chin. Pharm. Sci..

[79] Vecino X., Cruz J., Moldes A., Rodrigues L. (2017). Biosurfactants in cosmetic formulations: Trends and challenges. Crit. Rev. Biotechnol..

[80] Sarin Y., Kapahi B., Atal C. (1978). Scope of commercial utilization of some aromatic minor forest products from west Himalayas. Indian Perfum..

[81] Joshi R.K., Satyal P., Setzer W.N. (2016). Himalayan aromatic medicinal plants: A review of their ethnopharmacology, volatile phytochemistry, and biological activities. Medicines.

[82] Prakash P., Kumar M., Kumari N., Prakash S., Rathour S., Thakur M., Jamwal R., Janjua S., Ali M., Pundir A. (2021). Therapeutic uses of wild plants by rural inhabitants of Maraog region in district Shimla, Himachal pradesh, India. Horticulturae.

[83] Yang C., Hu D.H., Feng Y. (2015). Essential oil of *Artemisia vestita* exhibits potent in vitro and in vivo antibacterial activity: Investigation of the effect of oil on biofilm formation, leakage of potassium ions and survival curve measurement. Mol. Med. Rep..

[84] Sahoo B., Banik B. (2018). Medicinal plants: Source for immunosuppressive agents. Immunol. Curr. Res..

[85] Ding Y.-H., Wang H.-T., Shi S., Meng Y., Feng J.-C., Wu H.-B. (2019). Sesquiterpenoids from *Artemisia vestita* and their antifeedant and antifungal activities. Molecules.

[86] Irum S., Ahmed H., Mukhtar M., Mushtaq M., Mirza B., Donskow-Łysoniewska K., Qayyum M., Simsek S. (2015). Anthelmintic activity of *Artemisia vestita* Wall ex DC. and *Artemisia maritima* L. against *Haemonchuscontortus* from sheep. Vet. Parasitol..

[87] Sheng X., Sun Y., Yin Y., Chen T., Xu Q. (2008). Cirsilineol inhibits proliferation of cancer cells by inducing apoptosis via mitochondrial pathway. J. Pharm. Pharmacol..

[88] Sun Y., Wu X.-X., Yin Y., Gong F.-Y., Shen Y., Cai T.-T., Zhou X.-B., Wu X.-F., Xu Q. (2010). Novel immunomodulatory properties of cirsilineol through selective inhibition of IFN-γ signaling in a murine model of inflammatory bowel disease. Biochem. Pharmacol..

[89] Jung Y., Kim B., Ryu M.H., Kim H. (2018). Chinese medicines reported to have effects on contact dermatitis in the last 20 years. Chin. J. Integr. Med..

[90] Uniyal B., Shiva V. (2005). Traditional Knowledge on Medicinal Plants among Rural Women of the Garhwal Himalaya, Uttaranchal. Indian J. Tradit. Knowl..

[91] Frohlich E. (1968). Lavender oil; review of clinical, pharmacological and bacteriological studies. Contribution to clarification of the mechanism of action. Wien. Med..

[92] Rantzsch U., Vacca G., Dück R., Gillissen A. (2009). Anti-inflammatory effects of Myrtol standardized and other essential oils on alveolar macrophages from patients with chronic obstructive pulmonary disease. Eur. J. Med. Res..

[93] Rana D., Bhatt A., Lal B., Parkash O., Kumar A., Uniyal S.K. (2021). Use of medicinal plants for treating different ailments by the indigenous people of Churah subdivision of district Chamba, Himachal Pradesh, India. Environ. Dev. Sustain..

[94] Nin S., Arfaioli P., Bosetto M. (1995). Quantitative determination of some essential oil components of selected *Artemisia absinthium* plants. J. Essent. Oil Res..

[95] Oyedeji A.O., Afolayan A.J., Hutchings A. (2009). Compositional variation of the essential oils of *Artemisia afra* Jacq. from three provinces in South Africa—A case study of its safety. Nat. Prod. Commun..

[96] He L., Wu Y., Lin L., Wang J., Wu Y., Chen Y., Yi Z., Liu M., Pang X. (2011). Hispidulin, a small flavonoid molecule, suppresses the angiogenesis and growth of human pancreatic cancer by targeting vascular endothelial growth factor receptor 2-mediated PI3K/Akt/mTOR signaling pathway. Cancer Sci..

[97] Choudhury P.N., Choudhury M.D., Bhattacharjee N., Malakar C. (2014). A Study of in-silico Docking and ADME/Tox Property of Known Flavonoid Molecule Hispidulin. Pursuits.

[98] Sticher O. (1977). Plant mono-, di-and sesquiterpenoids with pharmacological or therapeutical activity. New Nat. Prod. Plant Drugs Pharmacol. Biol. Ther. Act..

[99] Rozman V., Kalinovic I., Korunic Z. (2007). Toxicity of naturally occurring compounds of Lamiaceae and Lauraceae to three stored-product insects. J. Stored Prod. Res..

[100] Obeng-Ofori D., Reichmuth C.H., Bekele A.J., Hassanali A. (1998). Toxicity and protectant potential of camphor, a major component of essential oil of *Ocimumkilimandscharicum*, against four stored product beetles. Int. J. Pest Manag..

[101] Obeng-Ofori D., Reichmuth C., Bekele J., Hassanali A. (1997). Biological activity of 1,8 cineole, a major component of essential oil of *Ocimumkenyense* (Ayobangira) against stored product beetles. J. Appl. Entomol..

[102] Lee B.-H., Annis P.C., Choi W.-S. (2004). Fumigant toxicity of essential oils from the Myrtaceae family and 1, 8-cineole against 3 major stored-grain insects. J. Stored Prod. Res..

[103] Abdelgaleil S.A., Mohamed M.I., Badawy M.E., El-arami S.A. (2009). Fumigant and contact toxicities of monoterpenes to *Sitophilus oryzae* (L.) and *Tribolium castaneum* (Herbst) and their inhibitory effects on acetylcholinesterase activity. J. Chem. Ecol..

[104] Liu P., Liu X.-C., Dong H.-W., Liu Z.-L., Du S.-S., Deng Z.-W. (2012). Chemical composition and insecticidal activity of the essential oil of *Illicium pachyphyllum* fruits against two grain storage insects. Molecules.

[105] Foster S., Duke J.A. (1990). A Field Guide to Medicinal Plants: Eastern and Central North America.

[106] S Mann R., E Kaufman P. (2012). Natural product pesticides: Their development, delivery and use against insect vectors. Mini Rev. Org. Chem..

[107] Zhu X.X., Yang L., Li Y.J., Zhang D., Chen Y., Kostecká P., Kmoníčková E., Zídek Z. (2013). Effects of sesquiterpene, flavonoid and coumarin types of compounds from *Artemisia annua* L. on production of mediators of angiogenesis. Pharmacol. Rep..

[108] Wang J.-L., Li C.-S., Dun Z., Zhou H.-Y. (2006). Immunosuppressive effect of Tibetan medicine, *Artemisia vestita* Wall extract. J. Sichuan Univ. Med. Sci. Chin..

[109] Wang J., Sun Y., Li Y., Xu Q. (2005). Aqueous extract from aerial parts of *Artemisia vestita*, a traditional Tibetan medicine, reduces contact sensitivity in mice by down-regulating the activation, adhesion and metalloproteinase production of T lymphocytes. Int. Immunopharmacol..

